# Precise Point Positioning Algorithm for Pseudolite Combined with GNSS in a Constrained Observation Environment

**DOI:** 10.3390/s20041120

**Published:** 2020-02-18

**Authors:** Chuanzhen Sheng, Xingli Gan, Baoguo Yu, Jingkui Zhang

**Affiliations:** 1 State Key Laboratory of Satellite Navigation System and Equipment Technology, Shijiazhuang 050081, China; shengchuanzhen@163.com (C.S.); yubg@sina.cn (B.Y.); cetc_zjk@163.com (J.Z.); 2 The 54th Research Institute of China Electronics Technology Group Corporation, Shijiazhuang 050081, China

**Keywords:** GNSS/pseudolite precise point positioning, urban canyon, low-cost receiver, distributed pseudolite, ambiguity resolution and validation, high-precision time synchronization

## Abstract

In urban canyon environments, Global Navigation Satellite System (GNSS) satellites are heavily obstructed with frequent rise and fall and severe multi-path errors induced by signal reflection, making it difficult to acquire precise, continuous, and reliable positioning information. To meet imperative demands for high-precision positioning of public users in complex environments, like urban canyons, and to solve the problems for GNSS/pseudolite positioning under these circumstances, the Global Navigation Satellite System (GNSS) Precision Point Positioning (PPP) algorithm combined with a pseudolite (PLS) was introduced. The former problems with the pseudolite PPP technique with distributed pseudo-satellites, which relies heavily on known points for initiation and prerequisite for previous high-precision time synchronization, were solved by means of a real-time equivalent clock error estimation algorithm, ambiguity fixing, and validation method. Experiments based on a low-cost receiver were performed, and the results show that in a weak obstructed environment with low-density building where the number of GNSS satellites was greater than seven, the accuracy of pseudolite/GNSS PPP with fixed ambiguity was better than 0.15 m; when there were less than four GNSS satellites in severely obstructed circumstances, it was impossible to obtain position by GNSS alone, but with the support of a pseudolite, the accuracy of PPP was able to be better than 0.3 m. Even without GNSS, the accuracy of PPP could be better than 0.5 m with only four pseudolites. The pseudolite/GNSS PPP algorithm presented in this paper can effectively improve availability with less GNSS or even without GNSS in constrained environments, like urban canyons in cities.

## 1. Introduction

The Global Satellite Navigation System (GNSS) plays a leading role in outdoor positioning and is the basis of location services for public users with low-cost receivers. However, GNSS cannot provide a continuous, high-precision location service in constrained environments like urban canyons, because: GNSS satellites, which are obstructed by high-density and tall buildings, result in poor positioning performance or even positioning failure [1,2], serious electromagnetic interference, dramatically degraded signal power, and severe multi-path error caused by reflation as a result of mirror building or minor structures, leading to low-quality observation data, especially for low-cost receivers [1,3]. GNSS satellites, which are faced with a short period of signal obstruction when travelling through billboards above, suffer from instantaneous GNSS signal loss and re-tracking. These are the main reasons for the frequent rise and fall of satellites in GNSS carriers and pseudorange measurements. In conclusion, the facts mentioned above increase the challenge of obtaining available, reliable, and accurate GNSS high-precision positioning information, and the public users’ high-precision positioning service will degrade and even fail under these circumstances.

Fortunately, the pseudolite, which can generate and transit GNSS-like navigation signals [4,5] and has the advantage of flexible placement, low cost, and easy maintenance, is an essential supplement of GNSS and, recently, has been adopted in many areas like precision positioning, timing, and deformation monitoring of bridges [6]. For complex urban canyon environments, the addition of a pseudolite can significantly improve the availability of GNSS-like satellites and enhance the spatial geometry of GNSS [3], which is now a foundation of high-precision GNSS positioning [7,8,9,10]; therefore, the use of a pseudolite with good spatial distribution provides an important technique for high-precise positioning in a constrained environment. However, this distributed pseudolite heavily relies on highly precise time synchronization for every pseudolite [11], which requires the support of a complex time-synchronization device with dedicated manipulation; moreover, the larger multi-path error of the pseudorange and the ambiguity initiation require known coordinates with high precision, problems which urgently need to be solved [12,13].

In view of the problem of obtaining high-precision positioning technology with a distributed pseudolite combined with GNSS in complex environments for practical applications, like urban canyons, an innovative method induced from the concept of Precision Point Positioning (PPP) and RTK (Real Time Kinematic) technology was designed in this study [14,15,16,17]. In this method, the problem of time synchronization of GNSS/pseudolite was solved by real-time equivalent clock error estimation. This differential correction information is generated based on real-time observation data from a reference station and is disseminated to surrounding users. With this differential correction, the low-cost users can use the PPP algorithm to get their own position with high precision. In view of a noisier pseudolite pseudorange with significant multi-path error, only pseudolite carrier phase observations are adopted in precise positioning. Besides, in this study, the pseudolite ambiguity estimation and ambiguity resolution validation algorithm was improved, which solves the problem of ambiguity resolution needing previous known coordinates with high precision. Finally, high-precision positioning of distributed pseudolite combined with GNSS in an urban canyon was conducted with a low-cost receiver. 

## 2. Methodology

The mathematical basics for the GNSS/pseudolite measurement model, time-synchronization algorithm, position determination method, and ambiguity resolution method are presented in detail in this section.

### 2.1. GNSS/Pseudolite Measurement Model 

The GNSS receiver (reference stations or public user) receives navigation signals transmitted by GNSS at the same time. Afterwards, acquisition, tracking and demodulating, navigation messages, pseudorange, carrier phase, and Doppler measurement occur [8,18]. For public users equipped with low-cost GNSS receivers, who are single-frequency users with only the GNSS L1 and B1 pseudorange, carrier phase, and Doppler measurement, the measurement model can be expressed as follows:

(1) $$\rho_{m,s}^{i}={\tilde{\rho }}_{m,s}^{i}-\delta I_{m,s}^{i}+\delta T_{m,s}^{i}+c\left(\delta t_{s}^{i}-\delta t_{m,s}\right)+\varepsilon_{m,s\rho }^{i}$$

(2) $$\varphi_{m,s}^{i}={\tilde{\rho }}_{m,s}^{i}+\delta I_{m,s}^{i}+\delta T_{m,s}^{i}+c\left(\delta t_{s}^{i}-\delta t_{m,s}\right)+\lambda N_{m,s}^{i}+\varepsilon_{m,s\varphi }^{i}$$

(3) $$d_{m,s}^{i}={\dot{\tilde{\rho }}}_{m,s}^{i}-\delta {\dot{I}}_{m,s}^{i}+\delta {\dot{T}}_{m,s}^{i}+c\left(\delta f_{s}^{i}-\delta f_{m,s}\right)+\varepsilon_{m,sd}^{i}$$

where $\rho_{m,s}^{i}$, $\varphi_{m,s}^{i}$ and, $d_{m,s}^{i}$ are the pseudorange, carrier phase, and Doppler measurements of GNSS respectively; $\varepsilon_{m,s\rho }^{i}$, $\varepsilon_{m,s\varphi }^{i}$, and $\varepsilon_{m,sd}^{i}$ are the measurement errors of those measurements respectively; m, s, and i are the receiver index (reference station as b or public user as r), GNSS flag, and GNSS PRN index, respectively; ${\tilde{\rho }}_{m,s}^{i}$ and ${\dot{\tilde{\rho }}}_{m,s}^{i}$ are the geometric distance and change rate of the geometric distance between the receiver m and transmitting antenna i of the GNSS satellite, respectively; $\delta I_{m,s}^{i}$ and $\delta {\dot{I}}_{m,s}^{i}$ are the ionosphere delay and change rate of the ionosphere delay, respectively; $\delta T_{m,s}^{i}$ and $\delta {\dot{T}}_{m,s}^{i}$ are the troposphere delay and change rate of troposphere delay, respectively; c is the speed of light; $\delta t_{s}^{i}$ and $\delta t_{m,s}$ are the GNSS satellite clock error and receiver clock error, respectively; $\delta f_{s}^{i}$ and $\delta f_{m,s}$ are the GNSS satellite frequency error and receiver frequency error, respectively; λ and $N_{m,s}^{i}$ are the wavelength and integer ambiguity of the carrier phase of GNSS satellite i, respectively.

Similar to GNSS, the receiver can also get the pseudorange, carrier phase, and Doppler observation of a pseudolite, because the pseudorange of a pseudolite has a much larger multi-path error, so only the carrier phase and Doppler observation of a pseudolite are used. If the ionosphere and troposphere delay are neglected, the measurement model is as follows:

(4) $$\varphi_{m,l}^{i}={\tilde{\rho }}_{m,l}^{i}+c\left(\delta t_{l}^{i}-\delta t_{m,l}\right)+\lambda N_{m,l}^{i}+\varepsilon_{m,l\varphi }^{i}$$

(5) $$d_{m,l}^{i}={\dot{\tilde{\rho }}}_{m,l}^{i}+c\left(\delta f_{l}^{i}-\delta f_{m,l}\right)+\varepsilon_{m,ld}^{i}$$

where $\varphi_{m,l}^{i}$ and $d_{m,l}^{i}$ are the carrier phase and Doppler measurements of the pseudolite, respectively; $\varepsilon_{m,l\varphi }^{i}$ and $\varepsilon_{m,ld}^{i}$ are the measurement errors of the carrier phase and Doppler measurements, respectively; ${\tilde{\rho }}_{m,l}^{i}$ and ${\dot{\tilde{\rho }}}_{m,l}^{i}$ are the geometric distance and change rate of geometric distance between the receiver and transmitting antenna i of the pseudolite, respectively; l and i are the pseudolite flag and pseudolite PRN index, respectively; $\delta t_{l}^{i}$ and $\delta t_{m,l}$ are the pseudolite clock error and receiver clock error, respectively; $\delta f_{l}^{i}$ and $\delta f_{m,l}$ are the pseudolite frequency error and receiver frequency error, respectively; λ and $N_{m,l}^{i}$ are the wavelength and integer ambiguity of the carrier phase of pseudolite satellite i, respectively.

### 2.2. GNSS/Pseudolite Time Synchronization Method

Precise Point Positioning (PPP) technology of pseudolites combined with GNSS heavily relies on the time synchronization of a GNSS satellite and pseudolite, and the accuracy of this depends on the technique and algorithm adopted in time synchronization [12], which includes satellite clock error in the GNSS, the satellite clock error in the pseudolite, and the relative system time biases between the GNSS and pseudolite. With respect to system time biases, the public user can estimate the receiver clock error for the pseudolite and GNSS, respectively.

As for time synchronization for the GNSS or pseudolite, which is not solved by the public user, it can be designed to be solved by means of the equivalent clock error based on reference station observations. The equivalent clock error for the GNSS carrier phase and pseudorange can be estimated and disseminated as a differential GNSS correction. The pseudorange equivalent satellite clock error, which mainly consists of the GNSS receiver clock error, the un-modeled satellite clock error, and un-modeled other errors remaining, is estimated for every GNSS satellite based on the GNSS pseudorange. Similar to the pseudorange equivalent clock error, the same method is used for the carrier phase equivalent clock error, except that the initial ambiguity for the carrier phase is heavily correlated with the GNSS clock error, so it is treated as a component of the GNSS carrier phase equivalent satellite clock error. The method for this is illustrated in this chapter:

If treating the pseudorange equivalent clock error as $\delta t_{b,s}^{i}$ and the carrier phase equivalent clock error as $\delta t_{b,s\varphi }^{i}$,

(6) $$\delta t_{b,s\varphi }^{i}=\delta t_{s}^{i}+\frac{\lambda N_{b,s}^{i}}{c}.$$

Then, the GNSS measurement model for a reference station can be rewritten as follows:

(7) $$\rho_{b,s}^{i}={\tilde{\rho }}_{b,s}^{i}-\delta I_{b,s}^{i}+\delta T_{b,s}^{i}+c\left(\delta t_{b,s}^{i}-\delta t_{b,s}\right)+\varepsilon_{b,s\rho }^{i}$$

(8) $$\varphi_{b,s}^{i}={\tilde{\rho }}_{b,s}^{i}+\delta I_{b,s}^{i}+\delta T_{b,s}^{i}+c\left(\delta t_{b,s\varphi }^{i}-\delta t_{b,s}\right)+\varepsilon_{b,s\varphi }^{i}$$

where ${\tilde{\rho }}_{b,s}^{i}$ can be obtained from a known reference station position and GNSS satellite position based on broadcast navigation; $\delta I_{b,s}^{i}$ and $\delta T_{b,s}^{i}$ can be obtained from normal troposphere and ionosphere models, such as the Saastamonien and Klubuchar models. $\delta t_{b,s}^{i}$, $\delta t_{b,s}$, and $\delta t_{b,s\varphi }^{i}$ must be solved based on the measurement model above. However, the pseudorange and carrier phase equivalent clock errors for every GNSS satellite are impossible to estimate simultaneously because of the correlation between the GNSS receiver clock error and GNSS equivalent satellite clock error. Therefore, the GNSS reference satellite is selected, and its pseudorange equivalent satellite clock error is obtained and strongly constrained to the prior GNSS satellite clock error from broadcast navigation. The selection criterion for the GNSS reference satellite is the largest elevation angle.

Similar to GNSS, time synchronization for a pseudolite is solved by the equivalent phase satellite clock error estimation based on the pseudolite carrier phase measurement for a reference station. Also, the pseudolite carrier phase ambiguity is heavily correlated with the pseudolite receiver clock, so it can be treated as a component of the phase equivalent clock error, which is the sum of the carrier phase ambiguity and pseudolite satellite clock error. Then, the phase equivalent satellite clock error can be expressed as follows:

(9) $$\delta t_{b,l\varphi }^{i}=\delta t_{l}^{i}+\frac{\lambda N_{b,l}^{i}}{c}.$$

Therefore, the pseudolite measurement model for a reference station can be rewritten as follows:

(10) $$\varphi_{b,l}^{i}={\tilde{\rho }}_{b,l}^{i}+c\left(\delta t_{b,l\varphi }^{i}-\delta t_{b,l}\right)+\varepsilon_{b,l\varphi }^{i}$$

where ${\tilde{\rho }}_{b,l}^{i}$ can be obtained from a known reference station position and pseudolite position. Similar to the GNSS pseudorange equivalent clock error estimation, the selection criterion of the pseudolite for the reference satellite is a continuous observation arc, and the phase equivalent satellite clock error of this satellite is set to zero to avoid correlation between the pseudolite phase equivalent clock error and pseudolite receiver clock error.

### 2.3. GNSS/Pseudolite PPP Observation Equation

With the GNSS/pseudolite equivalent clock error disseminated by a reference station, public users can adopt PPP technology to get an accurate position with their own pseudorange and carrier phase measurements. The PPP observation equations for public users can be expressed as follows:

(11) $$\delta \rho_{r,s}^{i}=\rho_{r,s}^{i}+\delta I_{r,s}^{i}-\delta T_{r,s}^{i}-c\left(\delta t_{b,s}^{i}+\delta t_{s}^{i}\right)={\tilde{\rho }}_{r,s}^{i}-c\delta t_{r,s}+\varepsilon_{r,s\rho }^{i}$$

(12) $$\delta \varphi_{r,s}^{i}=\varphi_{r,s}^{i}+\delta I_{r,s}^{i}+\delta T_{r,s}^{i}+c\left(\delta t_{b,s\varphi }^{i}+\delta t_{s}^{i}\right)={\tilde{\rho }}_{r,s}^{i}-c\delta t_{r,s}+\lambda N_{r,s}^{i}+\varepsilon_{r,s\varphi }^{i}$$

(13) $$\delta \varphi_{r,l}^{i}=\varphi_{r,l}^{i}-c\delta t_{b,l\varphi }^{i}={\tilde{\rho }}_{r,l}^{i}-c\delta t_{r,l}+\lambda N_{r,l}^{i}+\varepsilon_{r,l\varphi }^{i}$$

where the known $\delta t_{b,s}^{i}$, $\delta t_{b,s\varphi }^{i}$, and $\delta t_{b,l\varphi }^{i}$ are the GNSS pseudorange equivalent clock error, the GNSS carrier phase equivalent clock error, and the pseudolite carrier phase equivalent clock error, respectively. $\delta I_{r,s}^{i}$ and $\delta T_{r,s}^{i}$ can be obtained from normal troposphere and ionosphere models, respectively. ${\tilde{\rho }}_{r,s}^{i}$ and ${\tilde{\rho }}_{r,s}^{i}$ can be expressed as follows:

(14) $$\begin{array}{ll} {\tilde{\rho }}_{r,k}^{i} & =\frac{(x_{r}-x_{k}^{i})\times x_{r}+(y_{u}-y_{k}^{i})\times y_{r}+(z_{u}-z_{k}^{i})\times z_{r}}{\sqrt{{\left(x_{r}-x_{k}^{i}\right)}^{2}+{\left(y_{r}-y_{k}^{i}\right)}^{2}+{\left(z_{r}-z_{k}^{i}\right)}^{2}}}\\ & =\left[\begin{array}{ccc} e_{x,k}^{i} & e_{y,k}^{i} & e_{z,k}^{i} \end{array}\right]\times \left[\begin{array}{c} x_{r}\\ y_{r}\\ z_{r} \end{array}\right] \end{array}$$

where *k* is the index flag for GNSS s or pseudolite l, respectively; $x_{r}$, $y_{r}$, and $z_{r}$ are the three-dimensional coordinates of the public user; $x_{k}^{i}$, $y_{k}^{i}$, and $z_{k}^{i}$ represent the three-dimensional position of the GNSS or pseudolite, which can be solved with four GNSS/pseudolite observations and parameter estimations; and $\left[\begin{array}{ccc} e_{x,k}^{i} & e_{y,k}^{i} & e_{z,k}^{i} \end{array}\right]$ is called a geometry matrix.

The GNSS/pseudolite observation equation of PPP for public users can be expressed as the following matrix form:

(15) $$\left[\begin{array}{cccc} e_{x,s}^{1} & e_{y,s}^{1} & e_{z,s}^{1} & \\ e_{x,s}^{1} & e_{y,s}^{1} & e_{z,s}^{1} & \\ \begin{array}{l} e_{x,l}^{1}\\ \ldots \end{array} & \begin{array}{l} e_{y,l}^{1}\\ \ldots \end{array} & \begin{array}{l} e_{y,l}^{1}\\ \ldots \end{array} & \\ e_{x,l}^{n} & e_{y,l}^{n} & e_{z,l}^{n} & \end{array}\begin{array}{cccccc} c & 0 & 0 & 0 & \ldots & 0\\ c & 0 & \lambda & 0 & \ldots & 0\\ 0 & c & 0 & \lambda & \ldots & 0\\ \ldots & \ldots & \ldots & \ldots & \ldots & \ldots \\ 0 & c & 0 & 0 & \ldots & \lambda \end{array}\right]\cdot \left[\begin{array}{c} x_{r}\\ y_{r}\\ z_{r}\\ \begin{array}{l} \delta t_{r,s}\\ \delta t_{r,l}\\ N_{r,s}^{1}\\ N_{r,l}^{1}\\ \ldots \\ N_{r,l}^{n} \end{array} \end{array}\right]=\left[\begin{array}{c} \delta \rho_{r,s}^{1}\\ \delta \varphi_{r,s}^{1}\\ \begin{array}{l} \delta \varphi_{r,l}^{1}\\ \ldots \end{array}\\ \delta \varphi_{r,l}^{i} \end{array}\right]-\left[\begin{array}{c} \varepsilon_{r,s\rho }^{1}\\ \varepsilon_{r,s\varphi }^{1}\\ \begin{array}{l} \varepsilon_{r,l\varphi }^{1}\\ \ldots \end{array}\\ \varepsilon_{r,l\varphi }^{i} \end{array}\right].$$

### 2.4. Integer Ambiguity Resolution and Validation 

Using the observation equation above, public users have difficulty getting their own precise position with only pseudolite carrier phase measurements as result of a lack of pseudolite pseudorange measurements, which includes a large multi-path error, To solve this problem, the KPI (Known Point Initialization [19,20,21]) method has been proposed to achieve integer ambiguity of the pseudolite in advance, and the public user has to wait for a minute at a known precise position, which is very inconvenient for public users in motion. Fortunately, with more than four GNSS satellites available, public users can easily get a precise position, which provides important a priori knowledge for integer ambiguity resolution of a pseudolite in a constrained environment, such as an urban canyon. 

In one word, integer ambiguity for a pseudolite is easily obtained with the support of more than four GNSS satellites available for GNSS/pseudolite PPP technology. Afterwards, the carrier phase measurements for the pseudolite can be treated as a precise range if no cycle slip occurs. Based on these carrier phase measurements, the pseudolite PPP is adopted based on more than four pseudolite carrier phase measurements, even if there is no GNSS satellite in a deep urban canyon, or GNNS/pseudolite PPP can be adopted based on few pseudolites if there are not enough GNSS satellites available. In this constrained environment, this addition of a pseudolite can greatly improve the availability and reliability of high-precise positioning.

As discussed above, GNSS/pseudolite PPP technology in urban canyons requires accurate integer ambiguity for the pseudolite in advance; thus, integrity ambiguity resolution and validation are the foundation of GNSS/pseudolite PPP, which can be illustrated as follows. The position of the user, GNSS float ambiguity, GNSS receiver clock error, pseudolite float ambiguity, and pseudolite receiver clock error can be obtained and converge with no less than four GNSS satellites after a few epochs, supposing the estimated float parameter $X_{f}$ and its covariance $Q_{f}$ are in matrix form as follows:

(16) $$X_{f}={\left(\begin{array}{cccc} r_{f}^{T} & \delta t_{f}^{T} & N_{f,s}^{T} & N_{f,l}^{T} \end{array}\right)}^{T}$$

$r_{f}$ is the matrix form of three-dimensional coordinates for a public user; $\delta t_{f}$ is the matrix form of the receiver clock error, which consists of the GNSS receiver clock error and the pseudolite receiver clock error; $N_{f,s}$ is the matrix form of the GNSS float ambiguity; $N_{f,l}$ is the matrix form of the pseudolite float ambiguity.

The integer ambiguity resolution for GNSS is determined with the float ambiguity of the GNSS satellite difference $N_{f,s}^{T}$ and its covariance matrix deduced from $Q_{k}$, which can be obtained based on the transform matrix G, as follows:

(17) $$G=\left[\begin{array}{cccc} I_{3\times 3} & 0 & 0 & 0\\ 0 & 0 & D & 0\\ 0 & 0 & 0 & D \end{array}\right]\;where\;\;D=\left[\begin{array}{cccc} 1 & -1 & \ldots & 0\\ 1 & \ldots & \ldots & \ldots \\ 1 & 0 & \ldots & -1 \end{array}\right]$$

(18) $$X_{k}=GX_{f}={\left(\begin{array}{ccc} r_{f}^{T} & N_{f,s}^{T} & N_{f,l}^{T} \end{array}\right)}^{T}\;Q_{k}=GQ_{f}G^{T}.\;$$

The LAMBDA (Least Square Ambiguity Decorrelation Adjustment) method is adopted for the integer ambiguity resolution for the GNSS, and the popular ratio test algorithm is used to validate its correction in addition to the chi-square test based on the residual of GNSS measurements, only if the candidate of the GNSS initial integer ambiguity that passes all of the tests mentioned above can be substituted for the GNSS float ambiguity to get a more accurate position. After a few GNSS fixed solutions are achieved, the integer ambiguity resolution and validation of ambiguity resolution for the pseudolite begin to be performed. Similar to the integer ambiguity validation of GNSS, the popular ratio test algorithm and chi-square test are adopted; moreover, the time difference of the initial integer ambiguity between the current and previous epoch is used to validate the reliability of the initial integer ambiguity of the pseudolite. Once the ambiguity of the pseudolite is determined correctly, it can be used as a known value with strong constraint for the next epoch.

## 3. Implementations and Evaluation

**Figure 1:**
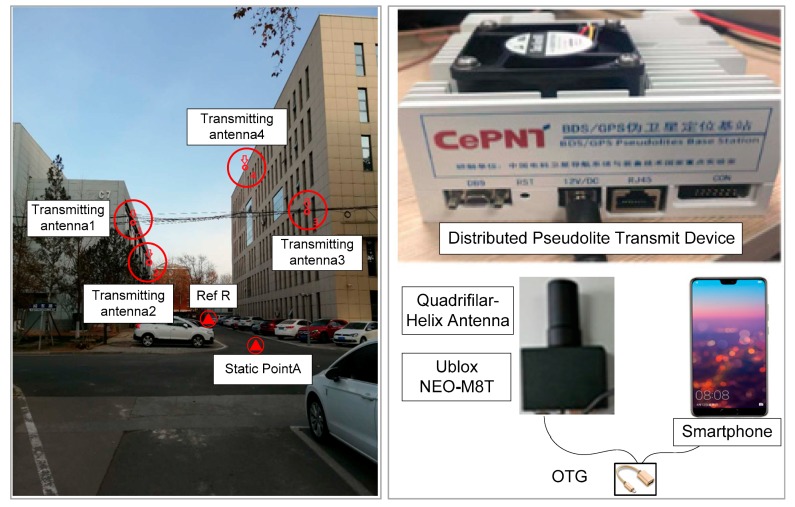
Experimental environments and setup.

**Figure 2:**
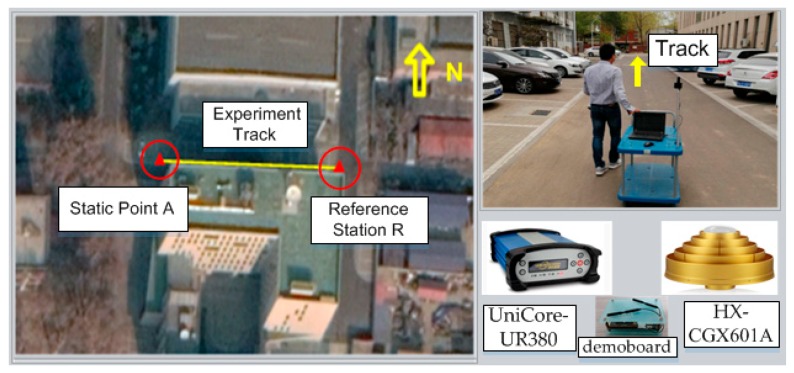
Kinematic test environment (experiment track shown with a yellow solid line).

In order to evaluate the performance of the Precise Point Positioning algorithm for a pseudolite combined with GNSS in a constrained observation environment, a GNSS/pseudolite PPP testbed was built in an urban canyon, as shown in Figure 1 and Figure 2. The experiment area was surrounded by a 16-meter-high building to the north and a 25-meter-high building to the south (Figure 2), so GNSS the satellite was heavily blocked; moreover, the surface of the surrounding building was doctored with mirror materials, which led to a serious multi-path error for the pseudorange in this area. Under these circumstances, there is a big risk for PPP to fail for public users with less than four GNSS satellite. Therefore, a distributed pseudolite system consisting of four pseudolites (from PL01 to PL04, shown in red circles) that can transit BDS (Beidou Navigation Satellite System) B1 frequency-like signals was deployed, and each transmitting antenna’s coordinate was measured in advance by a total station, as shown in Table 1. The size of the test area was about 80 meters long and 9 meters wide. The coordinates of static points for noise analysis were also measured by a total station in advance.

The reference station consisted of a HX-CGX601A GNSS choking ring antenna, and a UniCore-UR380 receiver modified to receive pseudolite signals was also deployed around the experiment field and marked in Google Maps [22] with a solid triangle as R (Figure 2). It was also equipped with a demoboard to obtain and disseminate an equivalent satellite clock error with 4G. A low-cost Ublox NEO-M8T receiver with a small quadrifilar-helix antenna was supposed to be used by users and was adopted in this experiment. In order to evaluate the noise of the GNSS and pseudolite in the urban canyon, the public user receiver was deployed at static point A with known coordinates; then, the kinematic test was performed to evaluate GNSS/pseudolite PPP when moving along the experiment track (yellow solid line marked in Figure 2). Finally, the PPP for public users was computed on a smartphone based on raw measurements from the Ublox receiver and equivalent satellite clock error received from the reference station. It is noteworthy that in the case of the equivalent satellite clock error being unavailable due to extreme conditions during 4G signal outages, these short-term faults during outages could be predicted using second-order polynomials by the smartphones of users.

### 3.1. Pseudolite Measurement Error Analysis 

A proper random model, which is vital for the GNSS/pseudolite PPP technique and important for data preprocessing, especially in urban canyons, needs a priori knowledge for the pseudorange and carrier phase noise. The pseudolite noise analysis was conducted based on a zero baseline and short baseline between the reference station and public receiver. For the zero baseline, the receivers of a public user and reference station used the same signal produced from the quadrifilar-helix antenna at point A. As for the short baseline, the reference station and user were deployed at static positions R and A, respectively, with known coordinates. After the double-difference geometry range between the receiver and satellite based on known coordinates was reduced from double-difference measurements of the pseudorange and carrier phase, the double-difference measurement residual error was obtained [23]; therefore, the characteristic of the pseudorange and carrier phase noise were analyzed for the zero baseline and short baseline based on those residual error measurements and are shown in Table 2 and Table 3 respectively:

The double-difference of the pseudorange and carrier phase for zero baseline cancels out the common error, so the remaining residual errors represent the noise level [24]. This shows that the noise of the pseudorange and carrier phase measurement are better than 0.2 m and 8 mm, respectively, and there is no significant system error.

For the short baseline, the double-difference residual errors are mainly multi-path errors except for measurement noise if we neglect other minor unmolded error. With consideration of the measurement noise from the zero baseline analysis, the multi-path error of the pseudorange can be larger than 3.472 m, with a maximum of 5.131 m (RMS, Root Mean Square). Relative to the multi-path error of the pseudorange, the multi-path error of carrier phase is not very large and the RMS of this is no larger than an 0.11 cycle. Therefore, if the pseudorange of the pseudolite is involved in the GNSS/pseudolite PPP technique, the pseudorange multipath will be absorbed by parameters, resulting in a worse position or divergence, so only the carrier phase is used in the GNSS/pseudolite PPP technique.

### 3.2. GNSS Measurement Error Analysis

**Figure 3:**
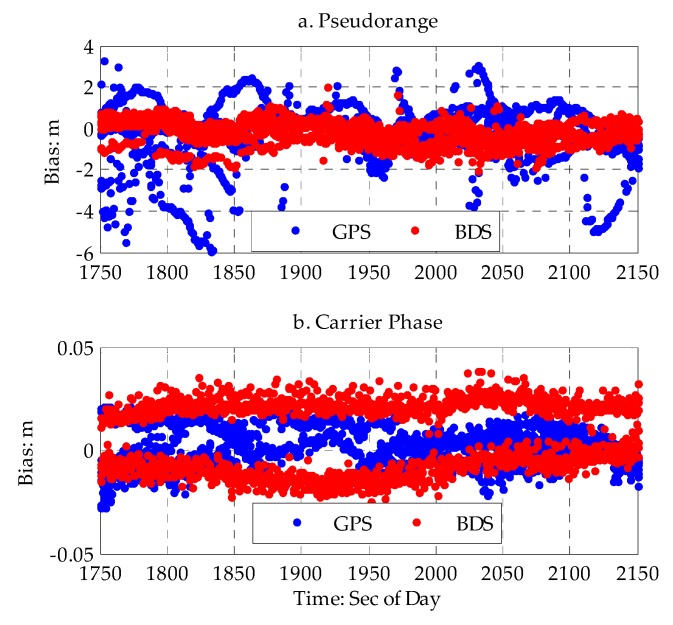
Time series of double-difference residual error for the pseudorange and carrier phase. (**a**) time series of double-difference residual error for pseudorange (top). (**b**) time series of double-difference residual of carrier phase (bottom).

In the urban canyon discussed above, the constrained and complex environment can deteriorate the performance of GNSS. Similar to the pseudolite measurement error analysis for a short baseline adopted above, the double-difference algorithm was adopted to analyze the measurement errors of GPS and BDS respectively. The time series of the double-difference residual error for GPS and BDS are shown in Figure 3 and Table 4.

The average (AVG) and root-mean-square (RMS) for the double-difference residual error were computed. The AVG and RMS for the double-difference residual error of the GPS pseudorange were −0.513 and 1.4567 m, respectively, which are nearly three times larger than the values in open space. The AVG and RMS for the double-difference residual error of the BDS pseudorange were −0.286 and 0.565 m, respectively. This shows that the measurement error of the pseudorange is much larger for urban canyons compared with good environments, and there are big biases and dispersion. The AVG and RMS for the double-difference residual error of the GPS carrier phase were 0.001 and 0.011 m, the AVG and RMS for the double-difference residual error of the BDS carrier phase were 0.01 and 0.015 m, and the measurement error of the carrier phase in the urban canyon was slightly larger than that in a good environment.

As discussed above, compared with a good environment, pseudorange errors of GPS or BDS are much larger in urban canyons; the large pseudorange error is more likely due to multi-path error as a result of GNSS signal refraction by the surrounding multi-mirror material, low-cost design of the Ublox receiver, and GNSS signal interference. It has a negative effect on the accuracy of PPP and integer ambiguity resolution.

### 3.3. Doppler-Based Cycle Slip Detection 

Carrier phase cycle slip detection is the basis of the GNSS/pseudolite PPP technique, especially for low-cost receivers with a cheap electronic component [10,25]. However, traditional cycle slip detection methods, such as the ionosphere residual error method and Melbourne–Wubeena combination, require two or three frequency measurements [26,27], so they not suitable for low-cost receivers with only a single frequency measurement. Fortunately, low-cost receivers can provide a pseudorange, carrier phase, and Doppler measurement. Thus, Doppler-based cycle slip detection is designed to solve the carrier phase cycle slip for low-cost receivers based on the carrier phase and Doppler measurement. The formula is expressed as follows:

(19) $$u=\left|\varphi_{m,s}^{i}\left(t+1\right)-\varphi_{m,s}^{i}\left(t\right)-d_{m,s}^{i}\right|\leq \eta$$

where *u* is the difference between the carrier phase difference and Doppler measurement; η is the threshold for Doppler-based cycle slip detection. Once difference u is larger than η, the corresponding satellite in the current epoch is marked as a cycle slip. The threshold of cycle-slip detection η is related to the accuracy of Doppler and carrier phase measurements. As for the pseudolite measurement error analysis above, the accuracy of the Doppler and carrier phase measurements are no more than 2.1 cm and 3 cm/s, respectively, so the cycle slip of a carrier phase larger than one cycle can be detected considering that one cycle is four times larger than a measurement error of 16.88 cm. In order to assess the performance of the method mentioned above, the PL01 and PL02 pseudolites were selected as examples. To compare with the Doppler-based cycle slip detection method, the time series of the carrier phase double-difference after deduction of the geometric distance between the satellite and a reference station/user with a known position is shown, and the epoch when cycle slip occurs is marked.

**Figure 4:**
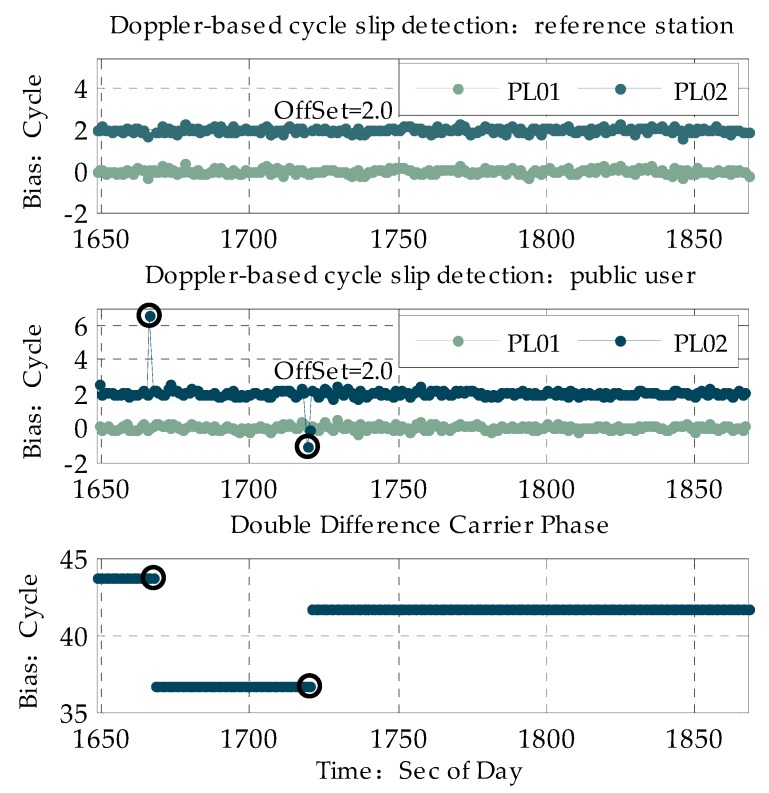
Cycle slip of PL01 and PL02.

From the carrier phase double-difference time series, it is shown that two cycle slips occur in Figure 4. These two cycle slips for the carrier phase are also detected based on the Doppler-based cycle slip detection method for the corresponding epoch and are marked as black circles. From Figure 4 above, we can also see that cycle slip only exists in pseudolite PL01 of the user and is detected by the Doppler-based cycle slip detection method with the un-difference measurement shown in the formula above; in other words, this cycle slip detection method has the obvious advantage that it can determine the exact pseudolite and exact receiver where cycle slip occurs, so this method is highly suitable for PPP technology with an un-difference measurement.

### 3.4. GNSS/Pseudolite PPP and Results

**Figure 5:**
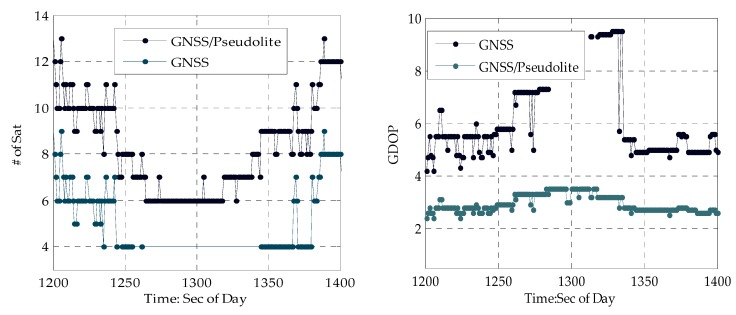
Time series of the number of satellites and DOP PDOP (Position Dilution of Precision).

A kinematic experiment along an experiment track (from A to R) was conducted, as depicted at the beginning of this chapter. The PDOP (Position Dilution of Precision) and the number of satellites available in this urban canyon are shown in Figure 5:

From Figure 5, it can be seen that when stepping from a normal urban canyon with low-density buildings to a deep urban canyon with high-density buildings, the number of GNSS satellites observed can decrease from nine to four, and the PDOP increases from 5.0 to 10.0 accordingly. When stepping into a terrible deep canyon with more high-density buildings around, PPP for users relying solely on GNSS fails when there are less than four GNSS satellites due to serious occlusion of GNSS signals by high-density buildings. When leaving the deep urban canyon, the number of GNSS satellites increases to nine, and PDOP decreases accordingly. Moreover, the number of GNSS satellites fluctuates greatly within a short time period, even in a normal urban canyon, so the GNSS PPP in an urban canyon is very difficult with frequency change of satellites and few GNSS satellites. Fortunately, the distributed-pseudolite provides continuous and sufficient GNSS-like satellites and solves the problem mentioned above. With four pseudolites in this area, the number of satellites increases to 13 and PDOP decreases to 2.2. Especially in the terrible deep canyon, accurate positions of public users can be obtained with four pseudolites available, as depicted in Figure 5 above.

**Figure 6:**
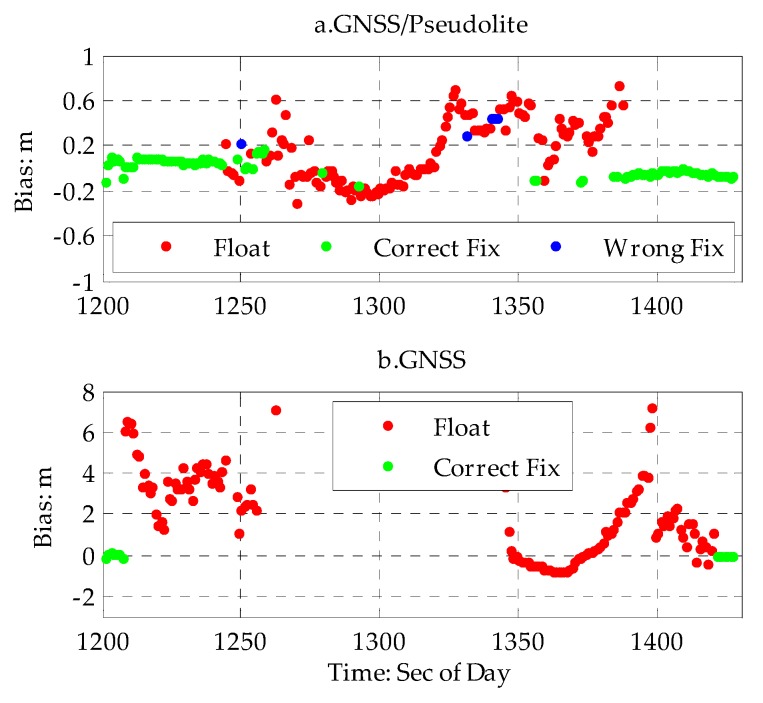
Time series of biases of height for the GNSS/pseudolite Precision Point Positioning (PPP) based on observation data. (**a**) time series biases of height for the GNSS/pseudolite PPP (top). (**b**) time series of biases of height for GNSS PPP (bottom).

The performance of the GNSS/pseudolite PPP was assessed based on kinematic experiment data as follows: PPP based on only GNSS and GNSS/pseudolite with the same date was assessed, respectively. The results are shown in Figure 6a,b, respectively. The position of the antenna of the low-cost receiver was determined using a smartphone with EKF (Extended Kalman Filter) following the GNSS/pseudolite PPP algorithm depicted in Section 2.3, and it relied on the raw pseudorange and carrier-phase measurements of the low-cost receiver and equivalent satellite clock error, which were estimated using LSE (Least Square Estimation) by a demoboard with raw data of the reference station, as depicted in Section 2.2, and this was disseminated by 4G. Before these estimations mentioned above, the cycle slip of carrier-phase had to be detected and marked with flags based on the Doppler-based cycle slip detection method depicted in Section 3.4 for the reference station in the demoboard and the low-cost receiver in the smartphone, respectively. Once the float-ambiguity solution was obtained, the ambiguity resolution and validation method for PPP were determined, as described in Section 2.4. For ambiguity resolution validation, the threshold for the numbers of satellites needed for ambiguity resolution and the ratio of ambiguity resolution were no less than four and three respectively. Moreover, in the GNSS/pseudolite PPP, only the pseudolite carrier phase was used due to the large multipath of the pseudorange. Unfortunately, it is difficult to get an accurate position for the kinematic reference track in an urban canyon, so only the height component of the position was selected to evaluate the PPP performance considering the small fluctuation height (less than 0.1 m) in the experiment area. Except for the all-solution and fixed-solution results, the proportions of fixed solutions and wrongly fixed solutions were also analyzed. The criterion for correctly fixed ambiguity was that the bias of the height of the fixed solution relative to the reference height was no more than 0.15 m.

As shown in Figure 6a, in a normal urban canyon with low-density buildings, the height accuracy of GNSS PPP with a higher proportion of ambiguity resolution can be better than 0.1 m as a result of there being enough GNSS satellites available, but it is not better than a few centimeters compared with a geodetic receiver in open space. The main reason for this is the low-cost design, larger multi-path error, as discussed in Section 3.2, and fluctuating satellites, as shown in Figure 5, which increase the risk of failure of ambiguity resolution. When entering a deep urban canyon with high-density buildings, the height accuracy of GNSS PPP with a low proportion of ambiguity resolution worsens greatly. The maximum (MAX) height bias is larger than 7.957 m as a result of there being less GNSS satellites available with heavy rise and upset. When stepping into a terrible deep canyon with more high-density building, GNSS PPP fails as result of there not being enough GNSS satellites. For nearly half of the experiment time, a precise position was not obtained with only GNSS satellites. Fortunately, the performance of GNSS PPP improved significantly with the help of the four distributed pseudolites available, and the height accuracy of the GNSS/pseudolite was better than 0.245 m (RMS), as shown in Figure 6b and Table 5, for the GNNS/pseudolite PPP. The proportion of fixed solutions increased to 47.7% and the height accuracy of the fixed solutions was better than 0.106 m. Meanwhile, the positions of the GNNS/pseudolite PPP with non-fixed solutions had smaller fluctuations, and the results were relatively reliable, as shown in Figure 6 above. The proportion of wrongly fixed solutions for the GNNS/pseudolite PPP was about 1.5%. The reason for this larger proportion of wrongly fixed solutions could be due to the larger multipath, frequency rise, and upset of GNSS satellites in the urban canyon.

Based on the analysis above, the performance of the GNSS/pseudolite PPP is greatly improved in urban canyons with four pseudolites available, especially in deep urban canyons with high-density buildings, where only the PPP with GNSS failed. Fortunately, with a continuous pseudolite carrier phase available, which has the advantage of having less loss of lock due to the higher-gain signal power transmitted by distributed pseudolites in the experiment field, highly accurate positioning information of public users based on the GNSS/pseudolite PPP is easy to get once the initial ambiguity of the pseudolite carrier phase is correctly determined. Afterwards, even if there is no GNSS satellite available, PPP with only four pseudolites can be used with the support of the initial integer ambiguity of the pseudolite determined previously. In order to illustrate the performance of PPP with only a pseudolite in a deep urban canyon with high-density building, an experiment was designed to simulate circumstances where there are only four pseudolites remaining when moving from a normal urban canyon to a deep urban canyon. The simulation data were based on GNSS/pseudolite observation data collected following the experiment trace (solid yellow line) in Figure 2 above.

**Figure 7:**
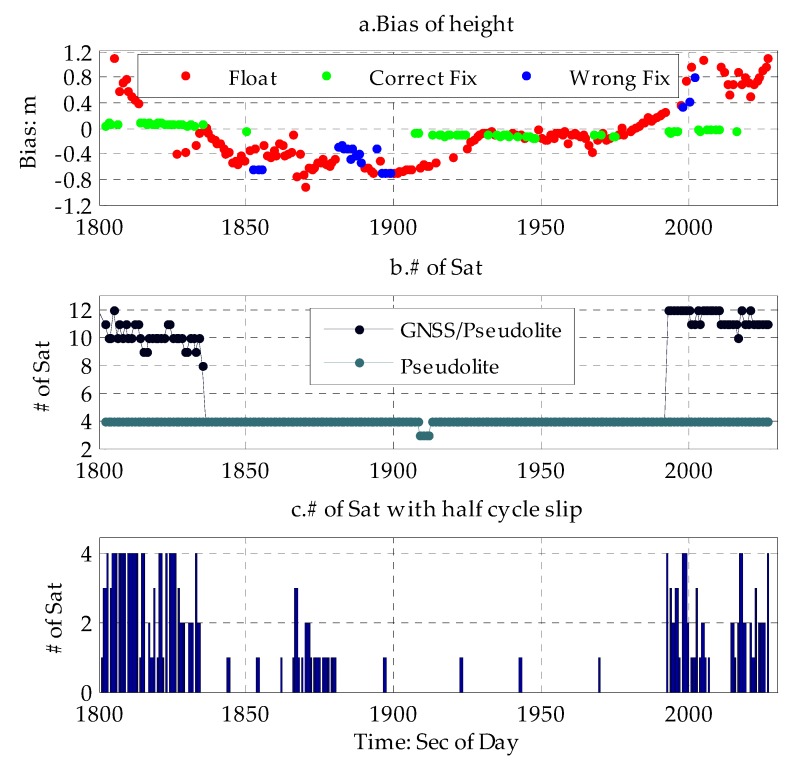
Time series of GNSS/pseudolite PPP based on simulation data. (**a**) time series of biases of height of GNSS/pseudolite PPP based on simulation data (top). (**b**) time series of the number of GNSS/pseudolite available (middle). (**c**) time series of number of satellite with half cycle slip (bottom).

As shown in Figure 7 and Table 6 bellow, when moving from a normal urban canyon with low-density buildings, the integer ambiguity of GNSS and pseudolites can be easily fixed by having more GNSS satellite available. Due to minor occultation of the GNSS, the accuracy of the GNSS/pseudolite PPP can be better than 0.1 m with the initial integer ambiguity of GNSS and pseudolite determined. This result is consistent with that discussed above. When entering a deep urban canyon with high-density buildings with the pseudolite intimal integer ambiguity determined previously, a situation was simulated where it was assumed that there were no GNSS satellites are available due to heavy occultation of the GNSS, but the carrier phase measurement of four pseudolite could be tracked continuously. The accuracy of PPP based on these four pseudolites can be better than 0.441 m with the previously-determined initial integer ambiguity of the pseudolites under strong constraint. Afterwards, even if the ambiguity resolution fails when the ambiguity resolution validation is rejected or half cycle slip of the carrier phase occurs, the maximum height biases of PPP with only four pseudolites are not larger than 1.0 m. Otherwise, the advantage for this method is that it does not require a known point for ambiguity initialization and is very suitable for constantly moving public users in urban canyons. The larger fluctuation for pseudolite PPP and the low proportion of fixed solutions shown in Figure 7 are heavily correlated with the half cycle slip of the carrier phase of the pseudolite marked by the receiver itself, and the poor geometry distribution is due to there being less pseudolites available.

## 4. Conclusions

GNSS PPP fails when there are few GNSS satellites in a deep urban canyon, where GNSS satellites are heavily obstructed by high-density building. Under these circumstances, distributed pseudolites are adopted to improve the availability and accuracy of GNSS/pseudolite PPP. The equivalent-clock error of the pseudorange and carrier phase were proposed to realize time synchronization of the pseudolite and GNSS, respectively. Afterwards, Doppler-based cycle slip detection for a Ublox single frequency receiver was proposed, and the multipath of the pseudorange and carrier phase measurement for GNSS/pseudolite were analyzed. The results showed that the multipath of the pseudorange for a pseudolite can be larger than 5.13 m; therefore, only carrier phase measurements are used in the GNSS/pseudolite PPP.

In a normal urban canyon with low-density building, the number of GNSS satellites that are slightly obstructed is no less than eight, and the accuracy of the GNSS/pseudolite PPP can be better than 0.15 m once the initial ambiguity has been correctly determined. However, when entering a deep urban canyon with high-density building, the number of GNSS satellites that are heavily obstructed decreases sharply and fluctuates greatly. The maximum biases for PPP with only GNSS satellites can be larger than 7.597 m. In extreme cases, the number of GNSS satellites is less than four, and with a larger pseudorange multipath, PPP with only GNSS satellite fails. In this situation, the accuracy of GNSS/pseudolite PPP can be better than 0.245 m. With the support of continuous pseudolite tracking, the stability and availability of PPP can be greatly improved; however, the accuracy of GNSS/pseudolite PPP is not better than a few centimeters. This is because the multi-path error of a low-cost receiver in an urban canyon with high-rise buildings is nearly three times larger than that in open space. The worse geometry distribution and satellites with frequent rise and fall in urban canyons, which increase the risk of failure of ambiguity resolution, are other two important factors that cannot be ignored in this constrained environment.

In an urban canyon with distributed pseudolites that are flexibly mounted at will and continuously tracked by a receiver, the number of GNSS/pseudolites available increases and the availability of GNSS/pseudolite PPP can be improved with the addition of pseudolite tracking. Dynamic experiments are designed based on observation field and simulation data; the results show that when entering a deep urban canyon with high-density building from a normal urban canyon with low-density building, the accuracy of GNSS/pseudolite PPP decreases greatly. In extreme cases, the accuracy of PPP can be better than 0.5 m with only four pseudolites with known integer ambiguity previously fixed. This method does not need a known point with high accuracy for initialization and is very suitable for public users in an urban canyon.

Cycle slip detection is a prerequisite for GNSS/pseudolite PPP. In order to solve cycle slip detection for low-cost PPP users, Doppler-based cycle slip detection has been proposed and it heavily depends on carrier phase noise, Doppler noise, and measurement sampling. Fortunately, most low-cost receivers have sampling rates better than 1 HZ and provide highly precise Doppler measurements. Considering that there were few clock jumps in the carrier phase in the receivers adopted in this paper, the effect of clock jump of the carrier phase on Doppler-based cycle slip detection could not be evaluated. The equivalent satellite clock error proposed in this paper for the pseudorange and carrier phase is not the exact satellite clock error among satellites, it also includes residual error for the un-modeled ionosphere and troposphere, and other errors. Besides the GNSS/pseudolite PPP with only GPS and BDS satellites was analyzed. With the addition of GALILEO, GLONASS, and other GNSS systems, the number of visible GNSS satellites would increase in urban canyons, and the accuracy of GNSS/pseudolite PPP would be further improved. Otherwise, integrated GNSS/INS/LidDAR-SLAM positioning is another valid technique for autonomous driving of vehicles in the case of GNSS failure, and it would be worth conducting research in this field to get high-precision positioning in urban canyons.

## Figures and Tables

**Table 1:** Coordinates of the four transmitting antennas.

	X	Y	Z
Transmitting antenna_1	−2080508.57	4578806.61	3909545.01
Transmitting antenna_2	−2080541.75	4578790.29	3909545.82
Transmitting antenna_3	−2080510.52	4578811.77	3909538.01
Transmitting antenna_4	−2080541.21	4578805.15	3909545.16

**Table 2:** Characteristics of pseudolite measurement error for zero baseline.

Satellite Pair	Pseudorange (meters)				Carrier Phase (cycle)			
	Max	Min	AVG	RMS	Max	Min	AVG	RMS
PL04-PL01	0.677	−0.699	0.008	0.195	0.012	−0.012	0.000	0.003
PL03-PL01	0.724	−0.605	0.060	0.183	0.013	−0.013	0.000	0.004
PL02-PL01	0.580	−0.698	−0.040	0.197	0.002	−0.033	−0.017	0.018
Mean	0.660	−0.667	0.009	0.192	0.009	−0.019	−0.006	0.008

**Table 3:** Characteristics of pseudolite measurement error for short baseline.

Satellite Pair	Pseudorange (meters)				Carrier Phase (cycle)			
	Max	Min	AVG	RMS	Max	Min	AVG	RMS
PL04-PL01	7.560	3.050	5.074	5.131	0.117	−0.143	0.080	0.080
PL03-PL01	−0.129	−3.642	−1.849	1.955	0.132	−0.227	0.090	0.093
PL02-PL01	−1.985	−4.552	−3.292	3.331	0.165	−0.198	0.099	0.102
Mean	1.815	−1.715	−0.022	3.472	0.138	−0.189	0.090	0.092

**Table 4:** Characteristics of Global Satellite Navigation System (GNSS) measurement error for short baseline.

.	Pseudorange (m)		Carrier Phase (m)	
	Average (AVG)	Root-Mean-Square (RMS)	AVG	RMS
GPS	−0.531	1.458	0.001	0.011
BDS (Beidou Navigation Satellite System)	−0.286	0.565	0.010	0.015

**Table 5:** Results of the height of the GNSS/pseudolite PPP based on observation data.

	All Solutions			Fixed Solutions			
	AVG	RMS	MAX	AVG	RMS	Fix	Wrong Fix
GNSS/Pseudolite (PLS)	0.093 m	0.245 m	0.735 m	0.014 m	0.106 m	47.7%	1.5%
GNSS	1.578 m	2.848 m	7.957 m	−0.049 m	0.085 m	7.6%	0.0%

**Table 6:** GNSS/pseudolite PPP results based on simulation data.

	All Solution			Fix Solution			
	AVG	RMS	MAX	AVG	RMS	Fix Ratio	Wrong Fix
GNSS/PLS	0.093 m	0.440 m	1.018 m	−0.089 m	0.269 m	34.7%	7.6%
